# Prevalence of pregnancy-relevant infections in a rural setting of Ghana

**DOI:** 10.1186/s12884-017-1351-3

**Published:** 2017-06-06

**Authors:** Fabian Völker, Paul Cooper, Oliver Bader, Angela Uy, Ortrud Zimmermann, Raimond Lugert, Uwe Groß

**Affiliations:** 1 Institute for Medical Microbiology, University Medical Center Göttingen, Kreuzbergring 57, D-37075 Göttingen, Germany; 2 St. Martin de Porres Hospital, Eikwe, W/R Ghana; 3 Göttingen International Health Network, Göttingen, Germany

**Keywords:** Pregnancy, Infections, Hepatitis B, Group B streptococci, Malaria, Ghana

## Abstract

**Background:**

Although infectious diseases still account for a high burden of morbidity and mortality in sub-Saharan Africa, simultaneous investigations on multiple infections affecting maternal and child health are missing.

**Methods:**

We conducted a cross-sectional, single-centre pilot study in a rural area of Ghana to assess the infectiological profile during pregnancy. Screening of 180 expectant mothers was done by vaginal swabs and serology to detect the most common pregnancy-relevant infections. They were also interviewed for potential risk factors, outcome of previous pregnancies, and socio-economic aspects.

**Results:**

We found a high prevalence of infections caused by hepatitis B virus (16.7% HBs antigen positive). In contrast, infections caused by hepatitis C virus (1.1% anti-HCV) and HIV (0.6%) were rare. Maternal malaria was frequent (10.6%), despite increasing acceptance of intermittent preventive treatment during pregnancy (IPTp). Group B streptococci were present in 10.6% of all pregnant women. Absence of antibodies against varicella zoster virus in 43.2%, *Toxoplasma gondii* in 26.8%, parvovirus B19 in 20.0%, and rubella virus in 15.7% makes a significant proportion of pregnant women susceptible for acquiring primary infections. Whereas all study participants had specific IgG antibodies against human cytomegalovirus, infections with *Listeria*, *Brucella*, or *Neisseria gonorrhoeae* as well as active syphilis were absent.

**Conclusions:**

Our pilot study in a rural community in Ghana indicates an urgent need for action in dealing at least with high-prevalent pregnancy-relevant infections, such as hepatitis B, malaria and those caused by group B streptococci. In addition, the resulting prevalence rates of various other infections may offer guidance for health officials to prioritize possible future intervention schemes.

**Electronic supplementary material:**

The online version of this article (doi:10.1186/s12884-017-1351-3) contains supplementary material, which is available to authorized users.

## Background

Between 1990 and 2015 the maternal mortality ratio in sub-Saharan Africa decreased from 987 maternal deaths per 100,000 live births to 546, but is still significantly higher than in high-income countries with only twelve deaths per 100,000 live births [1, 2]. The main medical causes for maternal death are hemorrhage, hypertensive disorders and infections which are accompanied by high mortality rates in low-income countries [3]. Concurrently, maternal health is closely linked to child health through the potential prenatal or perinatal transmission of pathogens. Hence, pregnancy-relevant infections present a major reason for high child morbidity and mortality rates in sub-Saharan Africa. Indeed, 1.2 million newborns die annually in sub-Saharan Africa with pregnancy and childbirth complications including childhood infections being among the five biggest challenges [4]. Despite pregnancy-relevant infections being an intensive field of research, their causes and possible co-infections are rarely specified on the pathogenic level in these countries. Only *Plasmodium* and HIV (co-) infections are often in the focus of research [5].

Based on this limitation in knowledge, the aim of our pilot study was to determine the prevalence rates of pregnancy-relevant infections which might have an impact on newborn health. We also aimed to evaluate the influence of socio-economic factors, such as access to medical services, age, origin and education on the prevalence of certain infectious diseases.

## Methods

### Study design

The study population consisted of pregnant women from the Jomoro, Nzema East Municipal, Ahanta West, and Ellembelle district (Western Region of Ghana) attending the hospital for delivery during time of investigation. The women had a gestational age of ≥39 weeks or an expected delivery within ≤1 week (independent of the week of pregnancy), hence including term and preterm deliveries. Non-hospitalized preterm birth could not be taken into account. A standardized questionnaire was used to collect general, demographic, and obstetric data (see Additional file 1). Specific antenatal medical care services were recorded. For data evaluation, we analyzed the participating women for accessibility of the hospital. Thus two sub-groups, one with relatively good access (origin: Ellembelle district, mean journey time 144 min) and one with limited access (origin: surrounding districts, mean journey time 229 min), were created.

Vaginal swabs were used to detect group B streptococci (GBS), *Listeria monocytogenes*, *Neisseria gonorrhoeae*, *Chlamydia trachomatis* as well as herpes simplex virus (HSV)1/−2. Blood/serum samples were analyzed for the presence of *Plasmodium species (spp.)* or antibodies against *Toxoplasma gondii*, *Treponema pallidum*, *Brucella spp*., rubella virus, varicella zoster virus (VZV), HSV1/−2, parvovirus B19 (PB19), human cytomegalovirus (HCMV), hepatitis B virus (HBV), hepatitis C virus (HCV), and HIV (combined detection of antigen and antibodies), respectively. Available HBs antigen (Ag)-positive serum samples were subsequently tested for HBeAg and elevated liver enzymes (alanine aminotransferase, ALT).

### Cultivation and identification of bacteria

Vaginal swabs were inoculated on blood and chocolate agar. Preliminary identification of bacteria was done at the local laboratory and subsequently confirmed by matrix-assisted laser desorption/ionization time-of-flight mass-spectrometry (MALDI-TOF MS) (MALDI Biotyper, Bruker Daltonics, Bremen, Germany) in Göttingen, Germany.

### Nucleic acid amplification

Real-time PCR was performed with the LightCycler 1.5 (Roche, Mannheim, Germany) to detect *C. trachomatis* and HSV in vaginal swabs. The Artus HSV-1/−2 LC PCR Kit (Qiagen, Hilden, Germany) was used to amplify HSV-1/−2 DNA. An in-house real-time PCR was applied for the detection of *C. trachomatis*. In brief, a 554 bp DNA fragment of the 16S rRNA gene was amplified using primers C161 (cgt cta ggc gga ttg aga g) and C162 (atc cac atc aag tat gca tcg) together with the LightCycler FastStart DNA Master SYBR Green I-Kit (Roche). Initial denaturation for 10 min at 95 °C was followed by 40 cycles of denaturation (10s, 95 °C), annealing (5 s, 60 °C) and elongation (20s, 72 °C).

### Malaria and serological tests

*Plasmodium* parasites were detected by peripheral blood smear and light microscopy using the Field’s stain technique. Serum samples were screened for pathogen-specific antibodies or antigens as listed in Table 1.

**Table 1:** List of applied serological assays

Object of assay	Test system	Producer
Toxo-IgM	VIDAS Toxo-IgM ELFA	bioMérieux, France
Toxo-IgG	VIDAS Toxo-IgG ELFA	bioMérieux, France
Toxo-IgG Avidity	VIDAS Toxo-IgG avidity ELFA	bioMérieux, France
HBsAg (HBV)	HBsAg(v2)/AXSYM	Abbott, Germany
HBsAg (HBV)	HBsAg EIA Test Kit	Ascon, USA (for comparison; rapid test with excellent performance used in Ghana)
HBeAg (HBV)	HBeAg(v2)/AXSYM	Abbott, Germany
Anti-HCV-IgG	HCV version 3.0 Anti-HCV	Abbott, Germany
Anti-HCMV-IgM/IgG	Cytomegalovirus IgM/IgG	Serion ELISA classic, Germany
Rubella IgM/IgG	Rubella virus (IgM/IgG)	Serion ELISA classic, Germany
HIV Ag/Ab	HIV Ag/Ab Combo	Abbott, Germany
Anti-PB19-IgM/IgG	Parvovirus B19 IgM/IgG	Mikrogen recomWell, Germany
Anti-VZV-IgA/IgG	Varicella-zoster virus (IgA/IgG)	Serion ELISA classic, Germany
Anti-HSV-IgM/IgG	Herpes simplex virus 1 + 2 IgM/IgG	Serion ELISA classic, Germany
*Treponema*-Ig	Serodia TPPA	Fujirebio, Japan
*T. pallidum*-Ig	FTA-ABS	Sekisui Diagnostics, Japan
Cardiolipin-Ig	VDRL	Omega Diagnostics, Germany

### Sample size estimation and statistical analysis

The sample size was estimated based on Kish’s formula [6]. A presumptive prevalence of 35% was estimated for most infections and used to maximize and get a reasonable number of samples to assess different infections in the target population. Characteristics of our study population were valued by using descriptive statistics. Chi square test was applied to compare categorical variables. A significance level of *P* < 0.05 was considered statistically significant. In order to estimate the impact of different independent variables that determine an outcome (e.g. seropositivity of a cohort) we used a logistic regression model. Data analysis was performed by using Stata 12 statistical software (www.stata.com).

## Results

### Study population and anamnesis

A total of 180 pregnant women (60% of the overall 300 deliveries during the study period) participated in the screening that took place in Eikwe/WR, Ghana during a three months period from October 2011 to January 2012. Emergency obstetric cases or deliveries during hospital night shift were excluded.

The mean age was 26.3 years (range 14–48 years). The average participant had her third pregnancy and less than two children. With up to 32%, the rate of illiteracy was high. The study population was divided into two subgroups according to the distance of living to the hospital which represented either good or limited access to medical care (*N* = 9 missing observations). Eighty-eight women were resident in the same district as the local hospital (subgroup 1) whereas 83 women had to travel from the surrounding districts (subgroup 2).

On average, an antenatal clinic (ANC) was visited 7.8 times during the current pregnancy. Based on the interview data, 83% received Fansidar as intermittent preventive treatment during pregnancy (IPTp), but only 34% were treated with three doses as recommended. Although guidelines in Ghana recommend screening for syphilis, hepatitis B and HIV during pregnancy, only 48% of the study population mentioned that they were screened for *T. pallidum* infection and only 17.2% were tested for HBsAg during their regular antenatal service. In contrast, 89% of pregnant woman had formerly received an HIV test.

### Prevalence of pregnancy-relevant infections

The results of direct pathogen detection or serologic determination of pathogen-specific antibodies are summarized in Table 2. According to the findings, the various pregnancy-relevant infections were classified into four statuses; (i) current infection, (ii) high proportion of women being susceptible for acquiring primary infection, (iii) past infection, and (iv) absence of infection.

**Table 2:** Prevalence of pathogens, pathogen-specific antigens and antibodies in the study population

Pathogen	*N*	Serological assays			Direct proof
		IgM	IgG	IgA	
Current pregnancy-relevant infections					
*HBV* ^*a*^ *(HBsAg pos)*	174	-	-	-	16·7%^e^
*Plasmodium* spp.^b^	180	-	-	-	+ 6·1% ++ 1·7% +++ 2·8%
*GBS* ^*c*^	180	-	-	-	10·6%
*HCV*	174	N/A	1·1%	N/A	-
*HIV*	173	0·6%*	0·6%*	0·6%*	-
*C. trachomatis* ^*d*^	177	-	-	-	1·7%
*HSV-1/−2*	174	11·5%	100%	N/A	1·1%^d^
High susceptibility for potentially acquiring primary infection					
VZV	169	n.d.	56·8%	2.4%	-
*T. gondii*	168	1·8%**	73·2%	n.d.	-
*Parvovirus B19*	170	4·7%	80·0%	N/A	-
*Rubella virus*	172	4·7%	84·3%	N/A	-
Past pregnancy-relevant infections					
CMV	172	0%	100%	N/A	-
*T. pallidum*	180	0%^+^	2·8 / 5·0%^++^	N/A	-
Absence of pregnancy-relevant infections					
N. gonorrhoeae^c^	180	-	-	-	0
*Listeria* spp.^c^	180	-	-	-	0

Direct pathogen proof was carried out by antigen test^a^, microscopy and counting^b^, cultivation^c^, or PCR^d^ . ^e^Five out of 23 tested HBsAg positive women (21.7%) were also HBeAg positive. -: not applicable. N/A: not available, n.d.: not done. *the assay used does not discriminate between antibody and antigen, **IgM-positive samples with high IgG avidity, ^+^VDRL, ^++^FTA-ABS/TPPA

### Prevalence of current infections

Serum samples from 174 women (*N* = 6 missing observations) were screened for HBsAg detecting a prevalence of 16.7% (*N* = 29). Five out of 23 available HBsAg-positive serum samples (21.7%) also tested positive for HBeAg. All of them had ALT values below cut-off levels indicating the absence of liver damage. Neither of the HBsAg-positive pregnant women received antiviral treatment, nor were they vaccinated against HBV. The prevalence of hepatitis B was considerably higher in subgroup 1 (21.8%) compared to subgroup 2 (11.2%; *p* < 0.05). However, none of the socio-economic factors were associated with HBsAg prevalence (data not shown). All HBsAg-positive mothers were HIV negative.

Only 1.1% (*N* = 2) of the entire study population had HCV-specific antibodies. Likewise, only 1 out of 173 (*N* = 7 missing observations) blood samples was tested positive for HIV-specific antibodies/antigens.

Screening of 180 blood samples detected *Plasmodium* parasites in 10.6% (*N* = 19) of the samples with eleven infected women harboring a low level, three a mid-level, and five a high level parasitemia.

Among the 180 study participants, a vaginal GBS carriage rate of 10.6% with the highest prevalence in the mean age group of 25–29 years (14.9%) was shown. However, study participants from subgroup 1 had significantly higher GBS colonization rates (15.9%) compared to pregnant women from subgroup 2 (6.0%; *p* < 0.01). According to our logit regression model, pregnant women from Ellembelle district had a 3-fold higher risk having a vaginal GBS colonization than women from other districts. Only three (1.7%) out of 177 vaginal specimens were tested positive for *C. trachomatis*.

Altogether 174 (*N* = 6 missing observations) women showed high levels of HSV IgG. Out of these, twenty (11.5%) also tested positive for HSV IgM. None of the study participants showed isolated elevated HSV-IgM titers. However, two (1.1%) out of 180 vaginal swabs were tested positive for HSV DNA, indicating active infection.

### High susceptibility for potentially acquiring primary infections

VZV IgA together with VZV IgG were detected in only 2.4% of 169 tested women (*N* = 11 missing observations), but 54.4% were positive for only VZV IgG. Since in total 56.8% were VZV seropositive, a large proportion (43.2%) was still potentially susceptible for acquiring primary infection. Neither maternal age nor the educational level was significantly different between seropositive and seronegative women.

The prevalence of *T. gondii* was determined serologically in 168 blood samples (*N* = 12 missing observations). 73.2% possessed specific *Toxoplasma* IgG, out of these, three had elevated levels of *Toxoplasma* IgM but high *Toxoplasma* IgG avidity ruling out infections within the last three to four months. Nevertheless, the absence of immunity in 26.8% of the women poses a risk for primary infection.

Altogether 80% of 170 women (*N* = 10 missing observations) were positive for PB19 IgG and 4.7% presented with PB19-IgM antibodies. A significant association existed between seropositivity and the number of pregnancies (*p* < 0.05); woman with >1 pregnancy had a 3.4-fold higher risk for being seropositive. However, based on our findings, 20% of the women were seronegative and thus susceptible for acquiring a primary PB19 infection.

Although none of the participants (*N* = 172, *N* = 8 missing observations) had received vaccination against rubella virus during childhood, high percentage (79.6%) of them were tested positive for rubella IgG without rubella IgM, which confirmed a subsided infection. A further 4.7% were seropositive for both IgM and IgG antibodies, indicating acute or more recently acquired infections. Absence of IgM and IgG antibodies in 15.7% identified a group that was susceptible for a primary rubella virus infection.

### Prevalence of past infections

None of 172 women (*N* = 8 missing observations) had HCMV IgM making it likely that acute HCMV infections were not present in the cohort. In contrast, all participants showed high HCMV IgG levels, indicating that they had at least partial immunity to HCMV.

All 180 study participants were screened for syphilis. Nine of them (5%) showed a positive *Treponema pallidum* particle agglutination (TPPA) result which indicated acute or past infections. Five (2.8%) were simultaneously tested positive by the fluorescent treponemal antibody absorption (FTA-ABS) test as well. Since none of them was tested positive by performing the veneral disease research laboratory (VDRL) test, we judged them as subsided syphilis infections. The individuals with an isolated-positive TPPA (*N* = 4) were probably false-positives due to immunological cross-reactions, e.g. with *T. pallidum ssp. pertenue* causing yaws which is endemic in Ghana.

### Absence of infections

In contrast to GBS, neither *L. monocytogenes* nor *N. gonorrhoeae* could be detected in any of the vaginal swabs. The same was true for *Brucella spp.* as determined by serology.

### Prevalence of coinfections

Co-infections were only found in pregnant women infected with HBV, the most prevalent current infection in our study sample. Several HBV-positive women were concurrently infected with GBS (1.1%) or showed serological signs of former contact with *T. pallidum* (0.6%).

## Discussion

This study provides information on the various infection statuses in the pregnant women in a rural Ghanaian community. Results may be biased by the not included women who do not receive antenatal care, or deliver at home. But according to local authorities this proportion is low on the basis of good medical education, relatively good transport connections of the surrounding communities and the introduction of a general health insurance (covers delivery costs) in 2003. In addition, good medical education may also explain on average 7.8 ANC visits which are far above what is usually seen in low-income settings.

With 10–17%, the most prevalent current pregnancy-relevant infections were hepatitis B, malaria, and vaginal GBS colonization. Although hepatitis B played the most important role as pregnancy-relevant infection, we found regional differences in HBsAg-carrier rates; a possible explanation for the high prevalence rate in subgroup 1 (21.8%) as compared to subgroup 2 (11.5%) may be its more remote location with less access to the public transport system and consequently lower risk of exposure. The overall high HBsAg prevalence of 16.7% was in agreement with other studies performed in urban areas of Ghana with prevalence rates of 10.6–16% [7, 8]. Also in other African countries with similar socio-economic and medical settings, such as Nigeria and Mali, HBsAg-carrier rate has been reported to be considerably high [9, 10]. Increasing age was not significantly associated with higher HBV prevalence rates in our cohort. This is in agreement with other studies which confirmed the majority of infections to occur in endemic areas by vertical maternofetal transmission [7]. The rate of maternofetal transmission is estimated to be 3–56%, depending on the maternal virus load [8]. More than 20% of the HBsAg-positive pregnant women in our study were also positive for HBeAg, indicating a high vertical transmission risk. Without vaccination or other medical intervention perinatal HBV infections lead to chronic progression in up to 90% of the affected newborns who are thus the source for later transmission to their own future offspring [11]. Consequently, efforts towards a gapless antenatal screening and a postnatal vaccination program for neonates of HBsAg-positive mothers need to be intensified in the future.

A substantial decline of placental malaria and maternal anemia with concurrent increase of the birthweight has been ascribed to the introduction of IPTp [12], which is a nationwide medical measure in Ghana since 2004. Although this malaria prophylaxis is well accepted among Ghanaian physicians, its complete implementation remains to be questionable; while 83% of the surveyed pregnant women of our study indeed received an IPTp, only 34% of those received the complete treatment with three doses. Thus, a large proportion of 66% was only preventively treated once or twice, limiting the effectiveness of IPTp. As a consequence, a malaria prevalence of 10.6% was found in our maternal population which is roughly in accordance with another study from Benin where a prevalence of 15.2% was found [13]. Thus, the completion of an entire IPTp scheme should be encouraged.

The observed 10.6% prevalence of vaginal GBS colonization was within the range of 4–25% shown for low-income countries [14]. Current data indicate a similar importance of GBS in neonatal sepsis with an even higher mortality rate in low-income countries compared to industrialized countries [15, 16]. Therefore, the role of GBS in neonatal sepsis in rural Ghana needs to be investigated more precisely in the future.

In contrast to the above mentioned infections, much less frequent with prevalence rates of only 0.6–1.7% were infections with HCV, HIV, *C. trachomati*s, and HSV. The anamnestic finding that 89% of pregnant woman had been routinely tested on HIV during antenatal care service is clearly an improvement over results from an earlier study in 2007 where only 12% of expectant mothers were tested nationwide. As a consequence of massive improvement of HIV/AIDS management in the last decades, only one HIV infection was identified in our cohort. The obtained data on HIV nearly equaled the 1.3% that was estimated in the 2013 sentinel survey in Ghana [17].

Although genital *C. trachomatis* infections are considered one of the most frequent STIs worldwide [18], its prevalence in Ghana seems to be low. The 1.7% prevalence rate of vaginal *C. trachomatis* colonization in our study population was similarly low as described for pregnant women in Accra (3.4%) [19].

The seroprevalence for HSV IgG was 100% in our study with 20 IgM-positive cases indicating a more recent HSV infection or reactivation during pregnancy. However, using real-time PCR, genital herpes was only detected in two cases (1.1%) which was nearly as low as a prevalence of 0.9% in Zimbabwe [20].

The absence of antibodies in a large proportion of pregnant women make them susceptible to primary infections, as is the case with VZV infection, toxoplasmosis, and infection with PB19 as well as rubella virus in our study cohort.

With 54.4%, the seroprevalence of VZV IgG in our cohort was significantly lower than that in industrialized countries, but in accordance with data from another study which indicated a higher seronegativity rate among immigrants from (sub-) tropical countries [21]. In combination with a non-existing VZV vaccine policy in Ghana, a lower natural virus circulation within the population might be the reason for this seroprevalence rate. Currently, a general VZV screening during pregnancy or selective testing of high-risk groups among pregnant women is not part of the clinical practice in Ghana. Furthermore, hospitals in rural areas do not have the means and resources to treat maternal or neonatal varicella. As a consequence, the incidence of maternal varicella and of congenital varicella syndrome is not known in Ghana.

Our study determined an IgG seroprevalence rate of 73.2% for *T. gondii*, including 1.1% of the participants who also tested IgM positive. Since these women presented high IgG avidities, an infection within the last few months was unlikely [22]. However, primary infection acquired during pregnancy could not completely be ruled out, since the screening was conducted in the very last month of pregnancy. Our data on seroprevalence of *Toxoplasma* IgG are in line with another recent study that showed a prevalence rate of 88.7% in pregnant women in Accra [23]. In contrast, a significantly lower rate of 20.3% was determined in Burkina Faso [24]. Whether this discrepancy is based on different test systems applied or different environmental burden with parasites remains to be clarified.

We demonstrated prevalence rates for PB19 IgM and IgG of 4.7% and 80%, respectively. Although this is in contrast to another study from sub-Saharan Africa with prevalence rates for PB19 IgM of 13.2% and IgG of 27.5% [25], absence of antibodies in 20% in our cohort makes them susceptible to primary infection. Similarly, we documented absence of antibodies against rubella virus in 15.7% of women in our cohort indicated that a significant proportion was susceptible to primary infection during pregnancy. This rate was twice as high as the one reported for Tanzania and argues in favor of a future vaccination program in low-endemic countries of sub-Saharan Africa [26].

The seroprevalence of HCMV IgG in our cohort was 100%, indicating past infection and making it unlikely that HCMV poses a major threat for prenatal transmission to the child in rural Ghana. Our results are significantly higher than the rate of 72.2% that was recently determined in Sudan [27]. Although a perinatal or postnatal HCMV transmission from IgG-seropositive mothers to their children is possible [28, 29], full-term infants are protected by maternal IgG in most cases and rarely develop symptoms.

Whereas no infection with *N. gonorrhoeae* could be identified, 5% had TPPA-reactive antibodies. Based on the results of the FTA-ABS and VDRL tests, we could not find active syphilis among our pregnant study participants. The TPPA results of our rural cohort matched with the outcome of another study performed in an urban Ghanaian community with a prevalence rate of 7.1% [19]. Finally, the absence of infections with *L. monocytogenes* and *Brucella* spp. might be based on the exclusive consumption of canned milk as only source of dairy products in our cohort.

## Conclusions

Mother and child morbidity and mortality rates in low-income countries are still among the highest in the world. Although non-communicable diseases are emerging in sub-Saharan Africa in the context of mother-child-health issues, infectious diseases play a major role. In Ghana, as in many other countries of sub-Saharan Africa, several diseases compete for the limited public health resources. Our pilot study indicates the existence of pregnancy-relevant infections in a rural population at different prevalence rates and indicates urgent need for action in dealing at least with those of high prevalence, such as hepatitis B, malaria and infections caused by group B streptococci. In addition, the resulting prevalence rates of various other infections may offer guidance for health officials to prioritize possible future intervention schemes.

## Additional file

Additional file 1: Structured questionnaire. Participants of the study were interviewed to collect general, demographic, and obstetric data using the structured questionnaire. (PDF 417 kb)

## Abbreviations

ALT: Alanine aminotransferase
ANC: Antenatal clinic
FTA-ABS: Fluorescent treponemal antibody absorption
GBS: Group B streptococci
HBeAg: Hepatitis B excretory antigen
HBsAg: Hepatitis B surface antigen
HBV: Hepatitis B virus
HCMV: Human cytomegalovirus
HCV: Hepatitis C virus
HIV: Human immunodeficiency virus
HSV: Herpes simplex virus
IPTp: Intermittent preventive treatment during pregnancy
MALDI-TOF: Matrix-assisted laser desorption/ionization time-of-flight
MS: Mass-spectrometry
PB19: Parvovirus B19
PCR: Polymerase chain reaction
spp.: Species
TPPA: *Treponema pallidum* particle assay
VDRL: Veneral disease research laboratory
VZV: Varicella zoster virus
