# Supplementary material for: Prevalence of pregnancy-relevant infections in a rural setting of Ghana
Source: BMC Pregnancy Childbirth. 2017 Jun 6;17:172. doi: 10.1186/s12884-017-1351-3 (PMC5460405; doi:10.1186/s12884-017-1351-3)
Supplement: Additional file 1: — Structured questionnaire. Participants of the study were interviewed to collect general, demographic, and obstetric data using the structured questionnaire. (PDF 417 kb) [file 12884_2017_1351_MOESM1_ESM.pdf]

Structured Questionnaire

Eikwe/Ghana risk factors during pregnancy

Q1. Name of home region ______________________________

Q2. Name of home district _______________________________

Q3. Name of home village ____________________________________

IF THE ANSWER TO ANY QUESTION IS 'DON'T KNOW', WRITE 97 NEXT TO THE CODES.
IF THE PERSON DOES NOT WANT TO ANSWER WRITE 98.

Demographic data

1. How many people live in your household? ______

2. How many adults (>17) live in your household? ______

3. How many children (<12) live in your household? ______

4. How many children younger than 5 years old live in your household? _______

5. Does your husband live in your household the whole year?
   1- Yes ☐ 2- No ☐

6. What was the highest education level you achieved? _________

7. Which vaccinations did the youngest children receive? **Use the card** CODING
   - ☐ BCG
   - ☐ OpV0
   - ☐ OpV1
   - ☐ OpV2
   - ☐ DPT1
   - ☐ DPT2
   - ☐ Others (please specify___________)

Nutrition

8. Do you use the water from the lake for drinking? 1- Yes ☐ 2-No ☐

9. How often did you eat meat during the last 7 days? Number ☐

10. What kind of meat?

- ☐ Chicken/Duck
- ☐ Goat
- ☐ Pork
- ☐ Beef
- ☐ Fish
- ☐ Bushmeat (please specify ________)
- ☐ Others (please specify___________)

11.How often did you drink milk during the last 7 days? Number ☐

12. How often did your children (< 5 years) drink milk during the last week?

Number ☐

13. Where is the milk coming from?

- ☐ 1- Directly from my own animals
- ☐ 2-Directly from animals of friends/neighbours
- ☐ 3- Unpacked milk from Shop
- ☐ 4- Packed milk from shop

14. If it is own milk, from neighbours/friends or unpacked is it mainly from?

- ☐ 1- a cow
- ☐ 2- a goat/sheep
- ☐ 3- not applicable

15. If it is own milk, from neighbours/friends or unpacked, how do you treat the milk before drinking?

- ☐ 1- Raw/Untreated
- ☐ 2- Heated/boiled
- ☐ 3- Fermented/Yoghurt

Health data ONLY Children < 12 years in your household

16	17	18	19	20	21	22	23	24	25	26	27	28
Child No.	Age	Place of birth	Is the child immunized against								Growth Rate	Age of mother at birth
			BCG	OPV			DPT			HeB		
				1	2	3	1	2	3			
1												
2												
3												
4												
5												
6												

1- at home without TBA

2- at home assited by TBA

3- Hospital Eikwe

4- other Hospital

1-Upper Red

2-Green

3-Lower Red

4-Grey

29. How often did you contact your health facility with your youngest child in the first year of life?

1. every month ☐
2. regularly ☐
3. only in case of illness ☐
4. never ☐
5. Don’t know ☐

30. How often did you contact your health facility with your youngest child afterwards until it reached the age of five?

1. every month ☐
2. regularly ☐
3. only in case of illness ☐
4. never ☐
5. Don’t know ☐

31. When your children fall ill, what do you do?

1.Self Treatment traditional medicine ☐
2.Traditional healer ☐
3.Drug store ☐
4.Health facility ☐
5.Other, specify ☐ _________________

32. Have any of the children in your household died before she/he reached the age of five?

1- Yes ☐ 2- No ☐ **if no Q39**

33. How many children have died? Number ☐

34. What symptoms did the child have before deaths?

1- Fever ☐
2- Diarrheal ☐
3- Cough ☐
4- Convulsion ☐
5. Fast and frequent breathing ☐
5- Other, specify ☐ _________________

36. How old was the child at deaths? _________ months OR _________ years

37. Did this child receive treatment before deaths of

1- No treatment ☐
2- Self treatment ☐
3-Health facility ☐
4-Traditional healer ☐
5- other specify _______________

38. How many days was the interval between onset of the disease and death? ____days

39. From your point of view what is the main problem in taking care of the health of your children?

____

40. How many of your children below the age of 5 slept under an impregnated bed net the last night? Number __________ (101 if no children below five)

Obstetric History Gravida __ / Para __

Fill in the Table with the respective codes given below!

41	42	43	44	45	46	47
Child No.	Year of birth	Mode of delivery	Place of delivery	Complications	Pregnancy outcome	Birth weight
				(multiple answers)		
1						
2						
3						
4						
5						
6						

Mode of delivery:

1. spontaneous vaginal delivery
2. vaginal operative delivery (Vacuum extraction)
3. primary caesarean section
4. secondary cesarean section

Place of delivery:

1- at home without TBA

2- at home assited by TBA

3- Eikwe Hospital

4- other Hopsital

3. APH (Ante Partum Hemorrhage)

4. PPH (Post Partum Hemorrhage)

5. Puerperal sepsis

6. Anemia

7. Malaria

8. Premature labour

9. Breech

10. Twins

11. Multiples

12. Asphyxia

13. other (please specify)

Pregnancy outcome:

1.live birth

2. still birth

3. neonatal death (within 28 days)

4. abortion (weeks of gestation)

48. In your last pregnancy, did you attend ANC? 1- Yes ☐ 2- No ☐

49. Are you pregnant at the moment of interview? 1- Yes ☐ 2- No ☐ if no Q53

50. Have you started attending ANC clinic (Ante Natal Care clinic)? Yes ☐ No ☐

51. If yes, how many months are you pregnant? _________ months

52. If not started ANC, when do you intend to start? __________ months

Have you been vaccinated against rubella?

Malaria treatment?

53. What are the things you like most in the ANC (Ante Natal Care clinic) clinic?

- ☐ Not attended
- ☐ Professionalism
- ☐ Safety
- ☐ Good for health
- ☐ Friendlyness
- ☐ Others (please specify___________)

54. What are the things you dislike in the ANC clinic?

- ☐ Costs
- ☐ Waiting
- ☐ Long distance to get there
- ☐ Others (please specify___________)

55. What do you think are the advantages of delivery at home?

- ☐ No Costs
- ☐ No Waiting
- ☐ Traditional
- ☐ Others (please specify___________)

56. What do you think are the advantages of delivery at the health facility?

- ☐ Safety
- ☐ Professionalism
- ☐ Easier
- ☐ Others (please specify___________)

58. Do you know any women, who died from complications of pregnancy within the last two years in your neighborhood? 1- Yes ☐ 2- No ☐ if No Q 62

59. How long ago did she die? Years ☐ Months ☐ Days ☐

60. If yes, where did she deliver?

at home without TBA	☐
at home assited by TBA	☐
Eikwe Hospital	☐
Other Hospital	☐
Other, specify	☐

61. If yes, what was the cause of death?

Complications:

1. Preeclampsia ☐
2. Eclampsia ☐
3. APH (Ante Partum Hemorrhage) ☐
4. PPH (Post Partum Hemorrhage) ☐
5. Puerperal sepsis ☐
6. Anemia in Pregnancy ☐
7. Malaria in Pregnancy ☐
8. Abortion complications ☐
9. Don't Know ☐
9. other (please specify) ☐

Transfusion Medicine

62. Do you suffer from anemia? 1- Yes ☐ 2-No ☐

63. If yes, are you aware of the diagnosis

☐ 1- Malaria
☐ 2- Parasitic worm infection
☐ 3- Sickle cell disease
☐ 4- Thalassaemia
☐ 5- iron deficiency
☐ 6- other (specify) ____________________

64. Have you ever received blood transfusion or is blood transfusion planned during the next days? 1- Yes ☐ 2-No ☐

65. If no, is this because of unavailability of blood donations? 1- Yes ☐ 2-No ☐

66. If yes, have you been asked for payment? 1 - Yes ☐ 2-No ☐

67. If yes, have you been asked for a replacement donation by a relative or friend?

1 - Yes ☐ 2-No ☐

Agricultural data

62. Which quality problems at harvest of crops (maize, beans, rice, cassava) do you know?

1- Insect damage ☐
2- Spoilage ☐
3- Fungi growth ☐
4- Discoloration ☐
3- Others (specify:______________________) ☐

63. What are the main quality problems during storage of crops above?

1- Insect damage ☐
2- Spoilage ☐
3- Fungi growth ☐
4- Discoloration ☐
5- Odour ☐
6- Others (specify:______________________) ☐

Water and Sanitation

64. What is your main water source for drinking? Only one option

☐ 1- Tap water (public)
☐ 2- Tap water on compound/in house
☐ 3- Public Pump
☐ 4- Well (protected at surface)
☐ 5- Well (unprotected at surface)
☐ 6- Lake
☐ 7- Rain water

65. Which type of latrine do the majority of members of your household use? Only one option

☐ 1- Pit latrine on site with flush
☐ 2- Pit latrine on site NO flush
☐ 3- Public Latrine
☐ 4- No latrine/Bush

66. Where do you dispose the stool of your children? Only one option

- ☐ 1- Near the house (forest/bush)
- ☐ 2- Far away from the house (forest/bush)
- ☐ 3- In latrine
- ☐ 4- Trash
- ☐ 5- Burried

67. Where do you wash yourself?

- ☐ 1-Lake
- ☐ 2- River
- ☐ 3- Pond
- ☐ 4- Home
- ☐ 5- Others (specify:______________________)

68. Where do you wash your children below the age of 5?

- ☐ 1- Lake
- ☐ 2- River
- ☐ 3- Pond
- ☐ 4- Home
- ☐ 5- Others (specify:______________________)

69. How many of your children <5 years had red urine now or during the last 4 weeks?

Number ☐

70. Do you know about the disease schistosomiasis (bilharzia)?

1- Yes ☐ 2- No ☐

Household characteristics

71. Does your household have electricity supply? 1- Yes ☐ 2- No ☐

72. Is the house you live in

- ☐ 1- Rented
- ☐ 2- Your own

73.Which of the following assets does your household posses

READ OUT THE ANSWERS AND WRITE DOWN THE NUMBERS!

1- Car		15- Juwlery	
2- Motorbike		16- Sewing Machine	
3- Bicycle		18- Chicken	
4- Boat		19- Ducks	
5- Radio		20- Pigs	
6- TV		21- Donkey	
7- Refrigerator		22- Goat	
8- Gas cooker		23- Sheep	
9- Fan		24- Cattle	
10- Mattress		25- Cats	
11- Beds		26- Dogs	
12- Table		27- Other Animals	
13- Chairs		28- Hand tools for working on the field	
14- Cell phone		29- Plough	
		30- Small Tractor	

74. Do your animals suffer from any visible disease?

☐ 1- Diarrhoea
☐ 2- Respiratory
☐ 3- Skin
☐ 4- Others (specify:______________________)

75. Does you or your husband have a bank account? 1- Yes ☐ 2-No ☐

Questionnaire Health Seeking Behavior:

ONLY FOR WOMEN WHO COME FROM OUTSIDE OF EIKWE!

Q1. Distance from the village to Eikwe (if only hours, write 0 days, number of hours, number of minutes):

	Days		Hours		Minutes

Q2. Mode of transport:

1	Car
2	Motorbike
3	Bike
4	By foot

Q3. Distance from home village to next delivery facility:

	Days		Hours		Minutes

Q4. Mode of transport:

1	Car/Bus
2	Motorbike
3	Bike
4	By foot

Q5. Why was this facility close to the home town not chosen for this birth?

Bad quality of facility (dirty, ….)	1	
Staff is not qualified	2	
Staff is rude	3	
Staff is corrupt	4	
Price for delivery too high	5	
Too expensive	6	
Coincidental (woman was close to Eikwe when labor pain started)	7	
Expected problems during birth	8	
Other	9	Specify here:________________________

Q6.Why was Eikwe hospital chosen?

Good reputation/image	1	
Trust in nuns	2	
Religious reasons (trust in god)	3	
Modern techniques (e.g. ultrasound)	4	
Fear, because of problems during pregnancy	5	
Friends recommend Eikwe	6	
Relatives recommend Eikwe	7	
Other	8	specify here:________________

Q.7 Who made the decision to go to Eikwe?

Woman who gives births herself	1	
Husband	2	
Mother of women	3	
Mother of husband	4	
Other family member	5	specify here: ____________________
Other	6	specify here: ____________________

Q8. How much did the transport cost from home town to Eikwe hospital?

[ ] Cedi

Q9. Why do other women from your village not go to Eikwe?

Too far	1	
Too expensive	2	
Husband does not allow	3	
They don't know Eikwe	4	
Other	5	specify here:_________________________

For women from Eikwe village

Q1.Why was Eikwe hospital chosen?

Good reputation/image	1	
Trust in nuns	2	
Religious reasons (trust in god)	3	
Modern techniques (e.g. ultrasound)	4	
Fear, because of problems during pregnancy	5	
Friends recommend hospital	6	
Relatives recommend hospital	7	
All women give birth there	8	
Other	9	specify here:____________________

Q.2 Who made the decision to go to Eikwe?

Woman who gives births herself	1	
Husband	2	
Mother of women	3	
Mother of husband	4	
Other family member	5	specify here: ____________________
Other	6	specify here: ____________________

Do you know women from Eikwe who give birth at home and not at the hospital?

Yes	1
No	2
Don't know	3
Don't want to answer	4
